# Unexpected improvement of hyperhidrosis with cannabidiol

**DOI:** 10.31744/einstein_journal/2022RC5795

**Published:** 2022-02-08

**Authors:** Rubens Pitliuk, Christina Morotomi Funatsu Coelho, Tatyanny Paula Pinto da Costa Santos Fucci

**Affiliations:** 1 Hospital Israelita Albert Einstein São Paulo SP Brazil Hospital Israelita Albert Einstein, São Paulo, SP, Brazil.; 2 São Paulo SP Brazil Neurologista, São Paulo, SP, Brazil.; 3 São Paulo SP Brazil Nutricionista, São Paulo, SP, Brazil.

**Keywords:** Cannabidiol, Dronabinol, Hyperhidrosis, Sweating, Phobia, social, Sympathectomy

## Abstract

Hyperhidrosis is characterized by excessive sweating and it affects almost 5% of the population. The affected age group is wide, and it can affect from children to elderlies. There are two types of hyperhidrosis: generalized and focal. Treatment depends on the symptoms presented. In more severe cases, radiofrequency sympatholysis and bilateral thoracic sympathectomy are the options. However, recurrence is possible or the postoperative appearance of conditions called compensatory hyperhidrosis or reflex hyperhidrosis. We describe two cases of patients treated with Cannabidiol who had significant and unexpected improvement of hyperhidrosis. The first patient received Cannabidiol specific for public presentations at work, and the second patient had a diagnosis of autism spectrum disorder. The hyperhidrosis improved in both patients immediately after using Cannabidiol.

## INTRODUCTION

Hyperhidrosis is a condition characterized by excessive sweating in some individuals. Sweat glands overreact to stimuli and they are usually overactive by producing more perspiration than physiologically necessary and for causing social, emotional, and professional harm. According to the International Hyperhidrosis Society, almost 5% of the world population suffers from excessive sweating or hyperhidrosis.^(1)^Hyperhidrosis can also affect children and adolescents and these individuals experience a considerably reduction in their quality of life.^(2)^

Patients with focal or primary hyperhidrosis often present with sweating involving the face, palms, soles of the feet, or armpits. Generalized sweating suggests secondary etiology. The most common causes of generalized sweating are excessive heat and obesity. Other causes include systemic diseases such as infections, endocrine disorders, neuroendocrine tumors, malignancy, neurological disorders, toxins, and previous spinal cord injuries. Unlike primary hyperhidrosis, patients with secondary generalized hyperhidrosis often present in adulthood and they have excessive sweating, which occurs both while awake and asleep.^(3)^

The diagnosis is strictly clinical. Treatment suggestions are made based on the location and severity of the case, and, in general, therapy is done with 20% topical aluminum chloride, botulinum toxin type A (Botox) and type B (Myobloc), and anticholinergic agents (glycopyrrolate, propantheline, and oxybutynin).

In patients with hyperhidrosis triggered by specific emotional events, current treatment options include: beta-blockers or benzodiazepines, iontophoresis, radiofrequency sympathicolysis, and bilateral thoracic sympathectomy (the gold standard treatment), done by videothoracoscopy, which should be recommended only in severe cases.

No clear difference has been demonstrated between the various techniques for bilateral thoracic sympathectomy. By achieving the correct level division, the results tend to be good and reproducible. The sympathetic chain can be sectioned, resected, removed with caution or divided with a harmonic scalpel, or clips can be used.^(3,4)^

There are reports describing compensatory hyperhidrosis and reflex hyperhidrosis postoperatively.^(5)^ In this article, we describe two cases of unexpected success using Cannabidiol (CBD) to control hyperhidrosis.

## CASE REPORT

### First case

A 43-year-old man who were social phobic since adolescence and had undergone numerous treatments such as psychotherapy, duloxetine, desvenlafaxine, paroxetine, clonazepam, escitalopram, propranolol, oxybutynin, quetiapine, valproate, aripiprazole, among other. Any of these therapies was totally effective, but all brought side effects, including increased sweating.

Before and during professional presentations, the patient felt tachycardia, dyspnea, extreme anxiety, and increased sweating, which made it difficult for him to perform at work. Treatment suggested was a one-time dose of 300mg of CBD without tetrahydrocannabinol, about 1 hour before going to his work.

To avoid the surprise of unwanted side effects at the time of the presentation, we recommended to the patient to take a 50mg test dose on a normal day at home with no public engagement.

Without any mention of hyperhidrosis (so there was no suggestion or placebo effect), he noticed that when greeting people, he no longer needed to dry his hands on a handkerchief that he kept in his pocket.

Many months later, he still had no hyperhidrosis. On his own, he stopped the CBD, and within a few days the hyperhidrosis returned. The CBD therapy was restarted, and the patient had improvement on the same day. He then maintains the 25mg dose in two takes (in the morning and at lunchtime) since September 2019.

### Second case

A 27-year-old woman diagnosed with autistic spectrum disorder, autonomic nervous system impairment, and epilepsy. Her additional complaints included multisegmental pain, anxiety, persistent thoughts, sweating on hands, difficulty in relating with people, and micturition discomfort characterized by frequent and urgent urge to urinate.

He had already used several medications, alone and in combination, without improvement of the complaints, and, many times, there was worsening of manifestations and/or new side effects. Among the medications already used were imipramine 75mg/day, oxybutynin 15mg/day, clonazepam 2.5mg/day, buspirone 5mg/day, atenolol 37.5mg/day, transcutaneous electrical stimulation (TENS) in pelvic floor (by recommendation of the urologist), oxcarbazepine 1,200mg/day, topiramate 200mg/day, lamotrigine 200mg/day, valproic acid 1,500mg/day, lacosamide 100mg/day, risperidone 2mg/day, and periciazine 20mg/day. The last combination therapy he had used was lamotrigine 200mg/day and risperidone 1mg/day, both with partial improvement of symptoms.

A combination of CBD (with up to 0.3% tetrahydrocannabinol) was suggested. The patient evolved with an important improvement in her complaints and, surprisingly, the sweating on her hands. The dose was titrated, starting at 10mg/day. The current dose used by patient is 50mg/day with maintenance of the basic medications. To date, the patient continues to use CBD.

This study was approved by the Research Ethics Committee of *Hospital Israelita Albert Einstein* under number # 4.571.353, CAAE: 43183221.6.0000.0071.

## DISCUSSION

Cannabinoids are used as alternative treatments in a wide range of diseases. The research on phytocannabinoids started in the last decades of the 20th century, in Israel.^(6)^ The modernization of laboratory techniques, starting in the 1960s, the isolation and structural elucidation of 9-tetrahydrocannabinol, and many other phytocannabinoids were possible.^(6,7)^

Cannabidiol has complex pharmacology, and several mechanisms have been proposed to explain its action. Different studies have shown that the acute effects of CBD clearly depend on the facilitation of neurotransmission mediated by the serotonin 5HT1A receptor in defense-related areas. In addition, CBD possesses a wide range of antipsychotic, sleep regulatory, antidepressant, anxiolytic, mood stabilizing, antiemetic, antiepileptic, anti-inflammatory, and analgesic properties.^(8-10)^

These reported cases demonstrated unexpected success in the control of hyperhidrosis and suggest the use of CBD as a new treatment possibility. This alternative treatment approach needs further investigations given that this was not extensively reported in the scientific published literature.
